# Osteopontin Impacts *West Nile virus* Pathogenesis and Resistance by Regulating Inflammasome Components and Cell Death in the Central Nervous System at Early Time Points

**DOI:** 10.1155/2017/7582437

**Published:** 2017-07-25

**Authors:** Nikki Bortell, Claudia Flynn, Bruno Conti, Howard S. Fox, Maria Cecilia G. Marcondes

**Affiliations:** ^1^Neurosciences Department, The Scripps Research Institute, La Jolla, San Diego, CA 92037, USA; ^2^Immunology and Microbial Science Department, The Scripps Research Institute, La Jolla, San Diego, CA 92037, USA; ^3^Department of Pharmacology and Experimental Neuroscience, University of Nebraska Medical Center, Omaha, NE 68198, USA; ^4^San Diego Biomedical Research Institute, San Diego, CA 92121, USA

## Abstract

Osteopontin (OPN) is a molecule that is common in central nervous system (CNS) pathologies, which participates in the activation, migration, and survival of inflammatory cells. However, the mechanisms by which OPN modulates inflammatory pathways are not clear. To understand the role of OPN in CNS viral infections, we used a lethal mouse model of *West Nile virus* (WNV), characterized by the injection of high doses of the Eg101 strain of WNV, causing the increase of OPN levels in the brain since early time points. To measure the impact of OPN in neuropathogenesis and resistance, we compared C57BI/6 WT with mice lacking the OPN gene (OPN KO). OPN KO presented a significantly higher mortality compared to WT mice, detectable since day 5 pi. Our data suggests that OPN expression at early time points may provide protection against viral spread in the CNS by negatively controlling the type I IFN-sensitive, caspase 1-dependent inflammasome, while promoting an alternative caspase 8-associated pathway, to control the apoptosis of infected cells during WNV infection in the CNS. Overall, we conclude that the expression of OPN maintains a critical threshold in the innate immune response that controls apoptosis and lethal viral spread in early CNS infection.

## 1. Introduction

The initial events following viral infection are crucial for pathogenesis and host outcome. This is especially true for rapidly replicating viruses, such as *West Nile virus* (WNV), in the central nervous system (CNS), where cells such as neurons are not replenished. *West Nile virus* (WNV), like other flaviviruses, is a single-stranded positive-sense RNA virus, which is carried by a mosquito vector and is transmitted to humans, birds, horses, and other animals. Originally found in Africa, Eastern Europe, and the Middle East, it was introduced into the United States in 1999. Since then, WNV has caused an outbreak of mosquito-borne neurological disease with the potential to induce a fatal form of viral encephalitis. While the infection of neurons is quite efficient, several other cell types can also host WNV replication. Two such cell types are macrophages and dendritic cells, which are likely responsible for the entry of the virus across the blood-brain barrier into the CNS [1, 2].

Both innate and adaptive immune responses play a role in controlling WNV infection while promoting repair in the CNS [3]. For instance, type I IFNs have been shown to be involved in protection against peripheral and CNS infection with WNV [4]. Furthermore, other proinflammatory components can initially clear a virus but can subsequently contribute to pathology [5]. However, the role of many of these proinflammatory molecules has not been defined. We have found that osteopontin (OPN) is a proinflammatory molecule that is increased in the brain early after infection with WNV, as well as in other CNS pathologies, such as multiple sclerosis, experimental autoimmune encephalitis, experimental stroke, simian immunodeficiency virus (SIV), and Theiler's virus infections [6–11].

Osteopontin (OPN) has been increasingly identified as an important regulator of both innate and adaptive immune responses in the CNS and elsewhere in the body [12]. OPN, an RGD-containing acidic glycoprotein, is one of the more highly secreted products of activated macrophages in inflammatory conditions [13] and in tissue repair processes [14]. Increased expression of OPN has been reported in pathological conditions, including those of the CNS; however, the role of OPN in viral encephalitis has been poorly explored.

Given its role in tissue repair and in cell migration [12], particularly in the CNS, we hypothesized that OPN is involved in the early host response to WNV. We therefore utilized a mouse model of WNV infection to address the participation of OPN at early time points in viral host interactions, CNS pathology, and subsequent survival. In animals injected with the median lethal dose (LD50) of the Eg101 WNV strain, we observed a protective role for OPN to increase mouse survival in correlation with controlled inflammasome and restricted apoptosis in the CNS.

## 2. Material and Methods

### 2.1. Virus, Animals, and Infection

A CNS-derived stock of WNV (Eg101 strain, obtained from Dr. M. Brinton) was produced by intraperitoneal inoculation of SCID mice with 10^3^ pfu of virus. Virus stocks were obtained from brain homogenates of SCID mice presenting disease symptoms and titrated using plaque assays on serial cell dilutions. For experimental analysis, B6.129S6 (Cg)-Spp1^tm1Blh^/J mice (OPN knockout) were obtained from the Jackson Labs (Bar Harbor, ME) and maintained at The Scripps Research Institute under pathogen-free conditions. For experimentation, mice (males, 6–8 weeks old) were infected intraperitoneally with 10^7^ pfu of the brain-derived stock. Plasma was obtained from the orbital plex prior to perfusion with ice cold PBS containing 10 mM EDTA. All experiments were performed with the approval of The Scripps Research Institute's Institutional Animal Care and Use Committee and Institutional Biosafety Committee and performed following NIH guidelines.

### 2.2. RNA Isolation and Quantitative Reverse Transcription PCR (qRT-PCR)

RNA was isolated from brain samples using TRIzol reagent (Invitrogen, Carlsbad, CA). cDNA was synthesized using a Superscript III kit (Invitrogen, Carlsbad, CA) as described (Yadav et al., 2007). The mRNA was quantified by quantitative reverse transcription PCR (qRT-PCR). The primers and probe sequences were designed using the Genescript online tool (https://www.genescript.com/ss-bin/app/primer) and obtained from IDT (San Diego, CA). Real-time reactions were performed in a 96-well plate in a Stratagene MX3000P (Stratagene, La Jolla, CA) using Platinum Supermix UDG (Invitrogen, CA) as previously described (Yadav et al., 2007). To compute the relative amounts of specific WNV mRNA in the samples, the average cycle threshold (Ct) of the primary cycle signal of the 18s ribosomal RNA (as control) was subtracted from that of the WNV to give the change in Ct (dct), which are log2 values. Viral titers were measured in the spleen and brain.

### 2.3. Plaque-Forming Unit (PFU) Assay

Confluent BHK cell monolayers in six-well culture plates were inoculated with 0.1 mL serial tenfold dilutions of WNV-containing material diluted in BA-1 diluent [M-199H, 1% bovine serum albumin, 0.05 M Tris (pH 7.6), 0.35 g sodium bicarbonate, 100 *μ*g penicillin, 100 *μ*g streptomycin sulfate, and 1 *μ*g fungizone]. Plates were incubated for 1 h at 37°C with 5% CO_2_. A primary overlay containing 0.6% agar in Eagle's minimal essential medium with 10% fetal bovine serum (FBS) was applied, and plates were incubated as described above. After a 48 h incubation, a second overlay containing 2% FBS and neutral red was applied to each well. Plates were returned to the incubator, and plaques were counted at 72 h postinfection. Plaque-forming unit assay and qRT-PCR correlation were tested from day 3 to day 7 pi in brains obtained from WT mice and correlated well with a resulting Pearson product-moment correlation coefficient of 0.95.

### 2.4. Immunohistochemistry

Mice were sacrificed upon perfusion with 0.2% EDTA-containing PBS while under isoflurane anesthesia. Brains were processed immediately after perfusion. Brains were removed and bisected in the midsagittal plane. The right hemisphere was snap frozen for RNA and/or plaque assay, and the left hemisphere was fixed in 10% formalin for immunohistochemical analysis. The following primary antibodies were used: WNV (Envelope protein; Abcam, Cambridge, MA), Iba-1 (Wako Chemicals, Richmond, VA), CD3 and Mac-3 (BD Biosciences, San Jose, CA), Fas (X-20, Santa Cruz Biotechnology, Dallas, TX), caspase 3 (Asp175, clone 5A1E, Cell Signaling, Danvers, MA), and PYCARD ASC-speck (MyBiosource, San Diego, CA). The antibodies were applied following a 40 min steam bath in 10 mM citrate buffer (pH 6.38) for antigen retrieval. Biotinylated secondary antibodies (Vector Labs, Burlingame, CA), followed by streptavidin HRP (Vector Labs), was developed by NovaRed (Vector Labs), and counterstaining was performed with Gill's hematoxylin (Sigma-Aldrich, St. Louis, MO). TUNEL staining was performed using the TdT in situ TACS Blue (R&D systems), following manufactures' instructions. Coverslips were applied over Cytoseal 60 Mounting Media (EMS, Hatfield, PA). Images were captured using an Axiovert 200 inverted microscope (Carl Zeiss) with Axio Vision software (version 4.8.1; Carl Zeiss). Image analysis was performed in Image J 6.4 (NIH, USA). For that, tiff image files were opened and manually thresholded to identify stained cells. A binary mask was obtained from the negative thresholded image, and measurement values were calculated as percentage of the total area.

### 2.5. Statistical Analysis

Two-way ANOVA followed by Bonferroni's test was used to identify significance in WT and OPN KO control groups, as well as 3 d and 5 d after WNV infection.

## 3. Results

### 3.1. OPN Delays WNV Entry into the Brain and Affects Animal Survival

**Figure 1:**
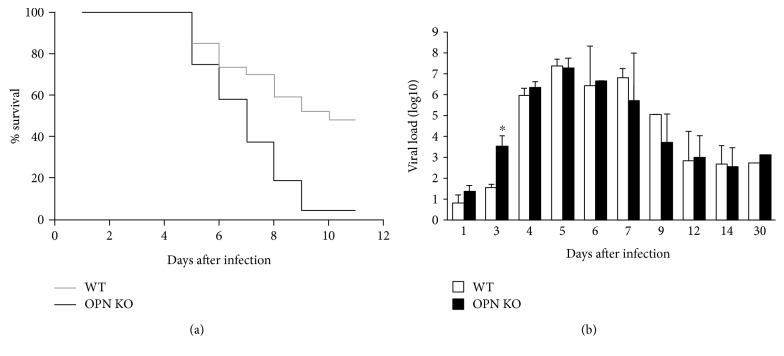
Survival and terminal brain viral load in WT and OPN KO animals. (a) Survival was assessed in WT (*n* = 27, gray line) and OPN KO animals (*n* = 16, black line) infected with 10^7^ pfu. (b) The viral load was evaluated in the brain of moribund animals prior to 9 dpi and of surviving animals after 9 dpi, both in WT (gray bars) and in OPN KO (black bars) mice. Results represent the mean ± SEM of 6 WT and 6 OPN KO mice. ^∗^*p* < 0.05, two-way ANOVA followed by Bonferroni's test.

The role of OPN in survival and viral entry was examined in wild-type (WT) and OPN knockout (OPN KO) mice by the intraperitoneal (ip) injection of a WNV Eg101 stock. As described, the Eg101 strain is milder in comparison to the broadly studied NY99 strain [15, 16], causing sickness symptoms, but not lethal, at low doses (10^4^ and 5 × 10^5^ pfu). However, inoculation with >5 × 10^6^ pfu led to morbidity and mortality. A 50% lethality rate was reached in WT mice following inoculation with 10^7^ pfu (LD50) and allowed for the comparison between WT animals and OPN KO. In spite of a significant 94% mortality detectable from day 5 postinfection (pi) (*p* value = 0.00031) (Figure 1(a)), OPN KO animals showed a significantly higher brain viral load only on day 3 pi, when compared to WT (*p* value = 0.00255) (Figure 1(b)). On day 5 pi, WNV levels in the brain were similar between WT and OPN KO mice (Figures 1(a) and 1(b)). This suggests that early time points are important in susceptibility and that OPN plays a role in host outcome. Low levels of virus (10^3^ pfu) were detectable in surviving animals from both groups at 30 dpi, suggesting that OPN does not affect persistence. In addition, viral load was consistently higher in the brain compared to peripheral sites such as spleen regardless of mouse background (>100-fold, data not shown), suggesting that OPN does not affect the CNS viral tropism. Given that early time points allowed detection of the differences in viral load and mortality, we focused on days 3 and 5 pi to investigate molecular mechanisms affected by OPN in vivo.

### 3.2. OPN Is Upregulated in the Brain of WNV-Infected WT Animals and Is Involved in Resistance to the Infection

**Figure 2:**
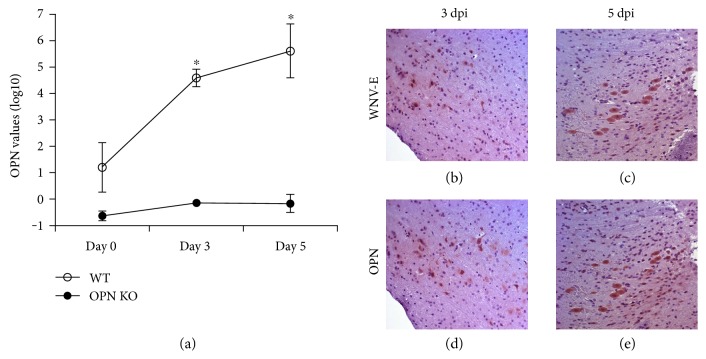
OPN levels correlate with WNV-infected cells in WT brains. Viral peptide WNV-E and OPN were detected in the brain of WT-infected animals 3 and 5 days following infection. (a) Detection of OPN transcripts in the brain of WT (open circles) and OPN KO (closed circles) animals at baseline, 3 and 5 dpi. Immunohistochemistry staining of WT animal brain frontal cortex for detection of WNV-E envelope (b) at 3 dpi and (c) at 5 dpi and of OPN protein (d) at 3 dpi and (e) at 5 dpi. Results represent the mean ± SEM of 6 animals per group. ^∗^*p* < 0.001, two-way ANOVA followed by Bonferroni's test.

We examined whether the infection induced the expression of OPN in the brain on days 3 and 5 pi. The ip inoculation of Eg101 LD50 significantly increased transcription of OPN in WT brains, since day 3 pi (*p* value = 0.002) (Figure 2(a)). By immunohistochemistry, we observed that virus (Figure 2(b)) and OPN protein (Figure 2(d)) were coexpressed at day 3. On day 5 pi, WNV (Figure 2(c)) and OPN-expressing WT cells (Figure 2(c)) showed morphological characteristics of neurons, as shown in frontal cortex sections. The expression of OPN in infected WT animals appeared to be closely correlated with sites of detectable virus, depicted by WNV envelope protein (WNV-E) (Figures 2(d) and 2(e)). This finding was consistent throughout the brain tissue (not shown). As expected, OPN was not detectable in the brains of OPN KO animals, at either day 3 or day 5 pi (not shown).

### 3.3. OPN Affects Acute Innate Immune Characteristics in WNV-Infected Brains

**Figure 3:**
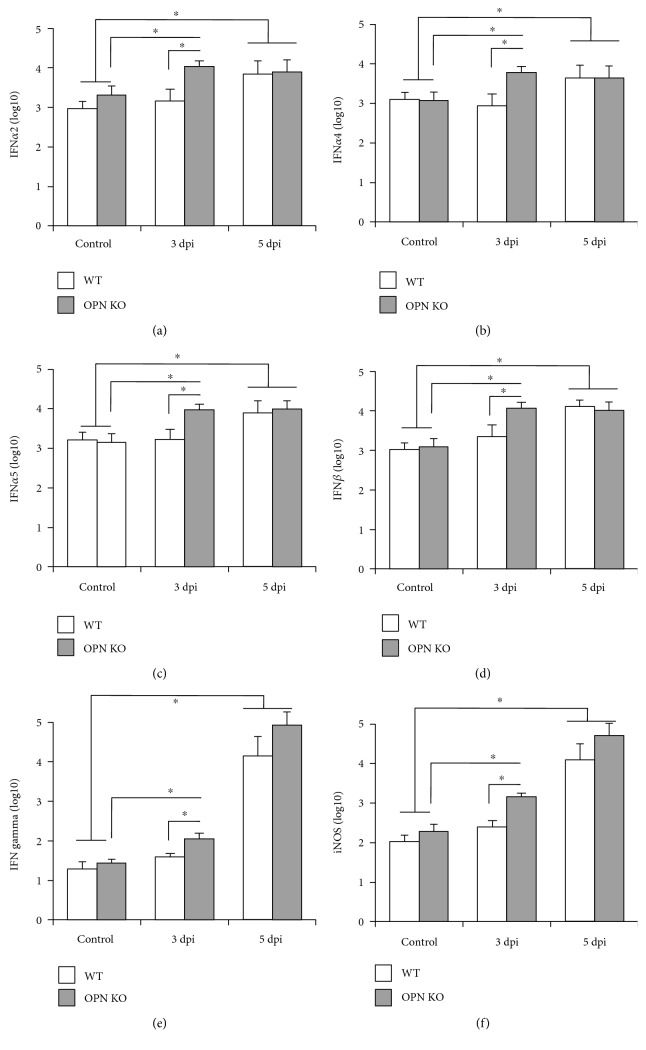
Innate immune and inflammatory cytokine transcriptional levels in the brains of WNV-infected mice. Cytokine levels were measured in the brains of perfused WT (white bars) and OPN KO (gray bars) animals, at baseline (control) and on days 3 and 5 pi. (a) INF alpha 2, (b) IFN alpha 4, (c) INF alpha 5, (d) INF beta, (e) IFN gamma, and (f) iNOS were measured by qRT-PCR. Results represent the mean ± SEM of 6 animals per group. ^∗^*p* < 0.001, two-way ANOVA followed by Bonferroni's multiple comparison test.

**Figure 4:**
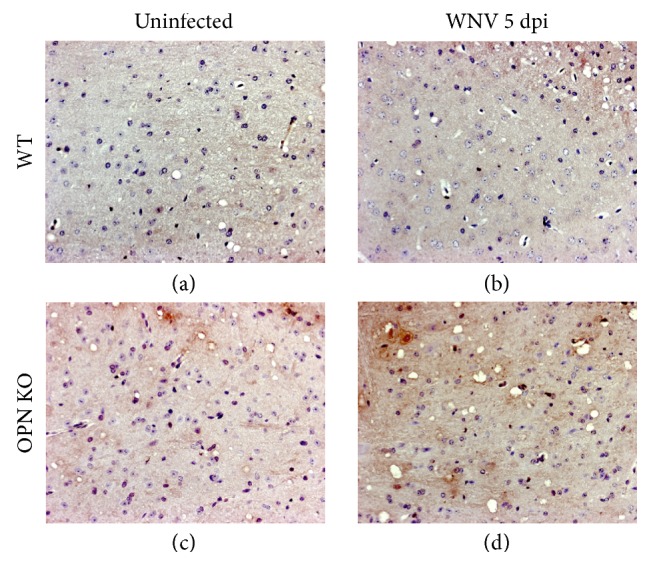
Detection of ASC-spec protein by immunohistochemistry in WNV-infected brains. Representative sections of frontal cortex from (a), (c) uninfected and (b), (d) WNV-infected animals at 5 day pi, (a), (b) WT and (c), (d) OPN KO, were examined for levels and distribution of ASC-spec protein using specific antibodies.

Given the role of innate immune responses, particularly type I interferons (IFN), at early time points in viral infections, we examined the hypothesis that OPN plays a role in modulating the levels of these cytokines in the brain. Baseline levels of type I IFNs (IFN*α*2, IFN*α*4, IFN*α*5, and IFN*β*) were similar between WTs and OPN KOs. Interestingly, OPN KO brains showed a faster upregulation of all the analyzed type I IFNs, which were detectable at day 3 pi, while WT brains increased type I IFNs only on day 5. A significant difference between WT and OPN KO on day 3 pi was observed for IFN*α*2 (*p* value = 0.0066) (Figure 3(a)), IFN*α*4 (*p* value = 0.0083) (Figure 3(b)), IFN*α*5 (*p* value = 0.018) (Figure 4(c)), and IFN*β* (*p* value = 0.006) (Figure 3(d)). IFN gamma and iNOS, which are also characteristics of the innate immune response, were also significantly elevated in the brain of WNV-infected OPN KO mice at day 3 after infection, compared to controls (*p* values = 0.007 and 0.0095, resp.) (Figures 3(e) and 3(f)), but levels were similar on day 5 pi. This data suggests OPN may negatively control the upregulation of innate immune genes at early time points following WNV infection in the brain.

### 3.4. OPN Affects the Caspase 1-Dependent Inflammasome in WNV-Infected Brains

Due to the differences in innate immune and proinflammatory genes, such as type I and II IFNs at day 3 pi, we hypothesized that OPN may also impact the expression of IFN-responsive genes in WNV-infected animals. Caspase 1 and other inflammasome molecules are highly responsive to IFNs, both positively and negatively [17–19]. We examined the levels of these molecules on day 5 pi, when differences in mortality were detectable.

**Figure 5:**
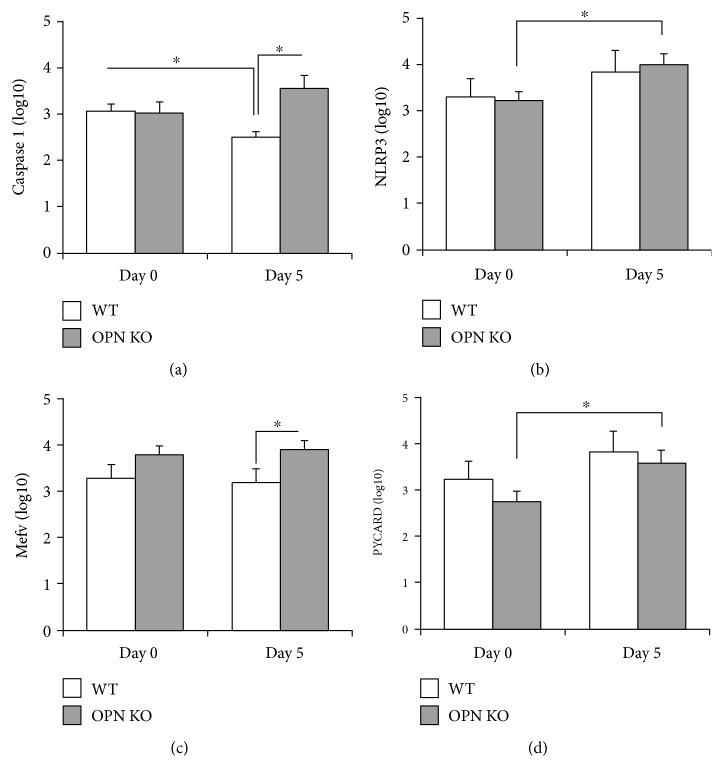
Transcription of inflammasome-associated molecules in WNV-infected brains. Transcriptional levels of (a) caspase 1, (b) NLRP3, (c) Mefv, and (d) PYCARD were measured by qRT-PCR, relative to GAPDH, on day 0 and day 5 post WNV infection, in WT (white bars) and in OPN KO (gray bars) brains. Results represent the mean ± SEM of 6 animals per group. ^∗^*p* < 0.05, lines indicate compared groups.

In our model, we found that compared to baseline, WNV-infected OPN KO brains had higher transcriptional levels of caspase 1 on day 5 pi (*p* value = 0.045), whereas WT brains showed decreased levels (*p* value = 0.047) (Figure 5(a)). Transcription of other inflammasome components, the NLR family pyrin domain containing 3 (NLRP3) and PYD and CARD domain containing (PYCARD) proteins, was significantly upregulated in OPN KO animals, on day 5 pi compared to baseline (*p* values = 0.02 and 0.024, resp.) (Figures 5(b) and 5(d)). Other inflammasome component, pyrin (Mefv), did not show a difference between baseline and day 5 pi. However, OPN KOs had significantly higher levels of Mefv when compared to WT on day 5 pi (*p* value = 0.048) (Figure 5(c)). These findings suggest that in the absence of OPN, the expression of inflammasome components in the brain is enhanced by the WNV infection.

**Figure 6:**
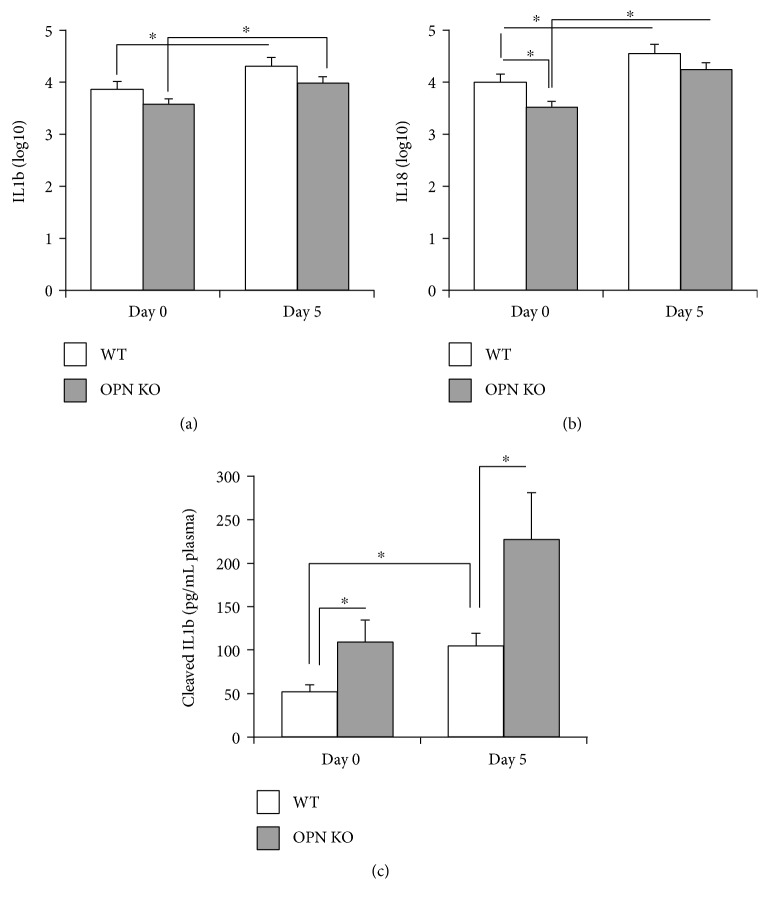
Transcription of IL1b and IL18 in the brain and cleaved IL1b in the plasma of WNV-infected brains. Transcriptional levels of (a) IL1b and (b) IL18 were measured by qRT-PCR, relative to GAPDH, on day 0 and day 5 post WNV infection, in WT (white bars) and in OPN KO (gray bars) brains. Results represent the mean ± SEM of 6 animals per group. ^∗^*p* < 0.05, lines indicate compared groups. (c) The mature form of IL1b was detected by ELISA in the plasma of WNV-infected WT (white bars) and OPN KO (gray bars), on days 0 and 5 after infection. Results represent the mean ± SEM of 3 control WT, 6 infected WT, 3 control OPN KO, and 6 infected OPN KO. ^∗^*p* < 0.05, lines indicate compared groups.

We further examined the ability of OPN to control inflammation in the brain after the infection, by measuring both upstream and downstream readouts of inflammasome activation. For instance, cells expressing the apoptosis-associated speck-like (ASC-speck) protein, which is required for the activation of caspase 1 [20], were more abundant in OPN KO mice, both at baseline and on day 5 pi compared to WT (Figure 4). Furthermore, the gene expression of IL1b and IL18, which are downstream molecules directly influenced by the inflammasome, were transcriptionally upregulated on day 5 pi in comparison with baseline values (Figures 6(a) and 6(b)). However, levels of circulating mature, cleaved IL1b were significantly enhanced in OPN KO in comparison to WT, both at baseline and at 5 dpi (Figure 6(c)), suggesting that OPN is indeed a negative regulator of the caspase 1-dependent inflammasome.

### 3.5. OPN Promotes a Caspase 1-Independent, Caspase 8-Mediated Inflammatory Pathway

**Figure 7:**
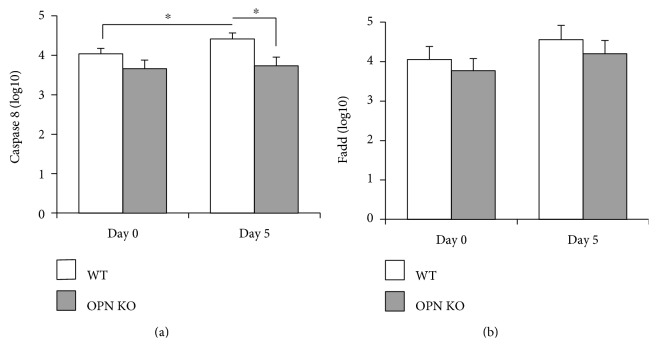
Transcription of caspase 8 and FADD in the brain tissue of WNV-infected brains. Transcriptional levels of (a) caspase 8 and (b) FADD were measured by qRT-PCR, relative to GAPDH, on day 0 and day 5 post WNV infection, in WT (white bars) and in OPN KO (gray bars) brains. Results represent the mean ± SEM of 6 animals per group. ^∗^*p* < 0.05, lines indicate compared groups.

We also examined the expression of components of an alternative, caspase 1-independent pathway, mediated by Fas and caspase 8. Here, we found that in WNV-infected WT mice at day 5 pi, OPN is necessary to upregulate caspase 8 (*p* value = 0.048) (Figure 7(a)), which may contribute to the increase in IL1b seen in WT animals (Figure 6(c)). The Fas-associated protein with death domain (FADD), another component of the caspase 8 inflammasome, transcriptional levels were not changed (Figure 7(b)). Thus, in WNV-infected brains, OPN may promote caspase 8.

### 3.6. OPN Prevents Cell Death in WNV-Infected Brains

**Figure 8:**
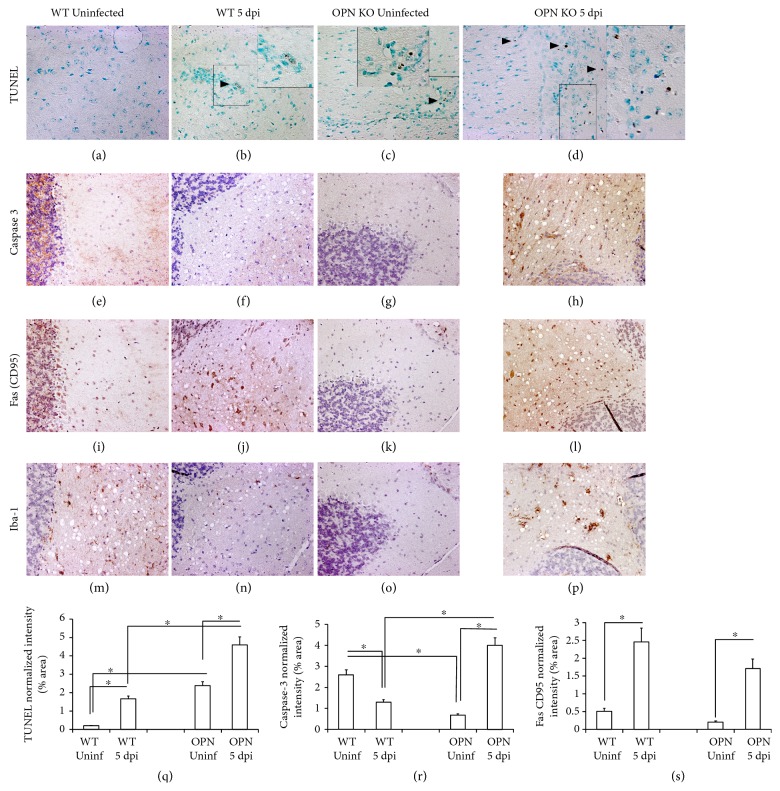
Immunohistochemical detection of apoptotic markers in WT and OPN KO mice infected with WNV. Representative sections of caudate obtained from WT and OPN KO animals showing the apoptosis marker TUNEL (a), (b), (c), (d). Representative serial sections of cerebellum obtained from WT and OPN KO mice, showing the effector caspase 3 (e), (f), (g), (h), Fas (CD95) expression (i), (j), (k), (l), and the microglia marker Iba-1 (m), (n), (o), (p). Markers were examined in uninfected WT (a), (e), (i), (m), infected WT brains at 5 dpi (b), (f), (j), (n), uninfected OPN KO (c), (g), (k), (o), and infected OPN KO brains at 5 dpi (d), (h), (l), (p). The intensity of the positive staining was calculated in thresholded whole brain images and normalized to the total brain area in ImageJ. Normalized density intensity of (q) TUNEL, (r) caspase 3, and (s) Fas (CD95). The values correspond to the average ± SEM of 6 animals/group. ^∗^*p* ≤ 0.05 in ANOVA comparisons followed by Bonferroni's post hoc tests.

We investigated the expression of proapoptotic molecules that were potentially affected by OPN in the brains of WNV-infected OPN KO mice. We examined TUNEL expression for the detection of apoptotic cells (Figures 8(a), 8(b), 8(c), and 8(d)), the effector caspase 3 (Figures 8(e), 8(f), 8(g), and 8(h)), Fas (CD95) (Figures 8(i), 8(j), 8(k), and 8(l)), and also the microglia activation marker Iba-1 (Figures 8(m), 8(n), 8(o), and 8(p)) in WT and OPN KO mice, control, and day 5-infected brains. As observed in representative sections of caudate, OPN KO animals showed a higher number of TUNEL+ cells (dark arrows) (Figure 8(d)) than infected WT mice (Figure 8(b)) both at baseline and on day 5 pi. In addition, we found that infected OPN KO mice had increased expression of caspase 3 protein (Figure 8(h)), while in WT mice, caspase 3 was decreased by infection (Figure 8(f)), as shown in representative sections of the cerebellum. The caspase 3-normalized intensity showed a correlation with TUNEL, as well as with Fas in OPN KO, but not in WT, on day 5 pi (Figures 8(g), 8(r), and 8(s)). However, Fas+ (CD95) staining was equally elevated in broth groups of animals after infection, in comparison to the baseline (Figures 8(i), 8(j), 8(k), 8(l), and 8(s)). Microglia activation was highly increased by infection in OPN KO mice, as revealed by a decreased Iba-1 staining on day 5 pi compared to uninfected and to WT animals (Figures 8(m), 8(n), 8(o), and 8(p)), in parallel with TUNEL and caspase 3, suggesting that microglial activation is associated with more cell death.

Taken together, these results indicate that OPN expression, particularly at early time points, provides protection against mechanisms of apoptosis that may favor the spread of the WNV infection in the CNS. This could be achieved by its ability to negatively control the type I IFN-induced inflammasome.

## 4. Discussion

Here, we have established that OPN has a protective role in a model of semilethal WNV infection, especially during acute and postacute time points. This early protective activity of OPN is exemplified by the premature increase in the brain, but not in peripheral, viral load in WNV-infected OPN KO mice. The acute differences in viral load resulted in a significantly higher mortality in OPN KOs compared to WT mice.

It is important to acknowledge that in our model, the dose of WNV Eg101 strain was elevated due to the mild capacity of this strain to induce mortality. The dose utilized here induced a 50% mortality rate in WT and allowed the distinction between the two mouse strain phenotypes. Although closely related, the lineage 1 clade 1a WNV Eg101 strain used here differs from the broadly studied NY99 strain, differing by 21 amino acid residues in the E sequence. This distinction causes Eg101 to be less neuroinvasive than NY99, due to the lack of an E protein glycosylation site [16], which may explain its milder effect. However, we observed a high number of infected cells in areas that are relevant for, and may replicate, human neurological outcomes resulting from the infection, such as the prefrontal cortex and cerebellum [21–23].

In our model, the mechanism by which OPN affected brain viral load, inflammation, and apoptosis may be through controlling the release of innate immune cytokines. Indeed, it has previously been shown that the expression of OPN has been associated with tissue repair in injury models [14]. OPN has been also shown to inhibit apoptosis in macrophages [24]. However, the role of this molecule in viral infections of the CNS is not clear.

To further investigate the mechanism for the relative resistance conferred by OPN, we investigated acute and postacute aspects of the IFN-mediated innate response. Our results are supported by findings that OPN can downregulate IFN gamma-induced iNOS expression [25]. Furthermore, it has also been shown that IFNg is important for controlling viremia as well as early brain infection [26]. Our results suggest that at early time points, OPN may sustain the IFNg levels for protection. The increased iNOS expression in OPN KO mice could also be involved in neuronal cytotoxicity, leading to enhanced viral spread and higher mortality [27]. Regarding type I IFNs, our results are supported by reports suggesting they are critical in host defense [28]. The IFN-mediated innate response components of the caspase 1 inflammasome, in particular, play important roles in inflammation and apoptosis.

**Figure 9:**
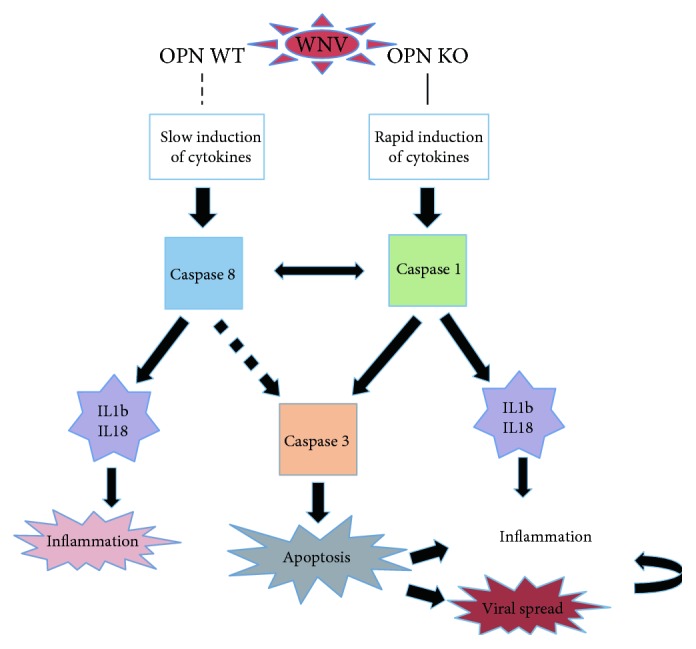
Schematic model for the role of OPN in WNV infection of the CNS. By delaying the induction of proinflammatory cytokines in vivo, OPN may act as a negative regulator of caspase 1-mediated apoptosis and IL1b, while promoting the induction of an alternative caspase 8 pathway. Conversely, in the absence of OPN, inflammatory cytokines are triggered soon after infection, activating caspase 1-mediated inflammasome components and ultimately resulting in an enrichment of caspase 3. As a result, cell death of infected cells may favor virus spread and further induction of secondary factors that aggravate the inflammatory pathogenesis in viral infections of the CNS, such as WNV.

It has been reported that in the absence of ASC-speck protein, which is encoded by the PYCARD gene and mediates inflammasome formation and caspase 1 activation, susceptibility to WNV neuropathogenesis is increased [29]. WNV-infected ASC KO mice have higher levels of inflammatory cells, with elevated IFN gamma, RANTES, and MCP-1, but lower levels of caspase 1 and IL1b [29]. This suggests the importance of inflammasome components in dealing with the virus in the CNS. In our model, transcription of PYCARD, as well as other inflammasome components, such as NLRP3 and caspase 1, was decreased by WNV infection in WT animals, while in OPN KO, those were increased. Thus, OPN KOs failed to regulate these components, as shown by the upregulation of caspase 1. The increase in caspase 3 in correlation with enhanced apoptotic TUNEL-positive cells in OPN KO mice suggests that this is a mechanism associated with susceptibility, likely due to facilitating viral spread by killing infected cells. On the other hand, in WT animals, a decreased expression of caspase 3 after infection may be associated with a lower level of apoptosis, compared to OPN KO mice. Therefore, OPN at early time points may regulate a threshold in the virus-controlling innate response, preventing encephalitis and apoptosis of infected cells.  Figure 9() shows a scheme of the mechanism we propose based on our findings.

A failure to increase caspase 3 at the protein level or perhaps a higher degradation of that molecule, in infected WT animals, is part of the mechanism that prevents a more drastic increase of apoptosis in WT animals. Importantly, CD95/Fas levels are higher in all infected mice compared to controls, regardless of background. Thus, the increase in TUNEL+ cells is higher in the brain of infected OPN KO, where both caspase 3 and CD95 are highly upregulated, when compared to WT animals, where caspase 3 is controlled and only CD95 is elevated by infection. This is in agreement with the proposed mechanism, summarized in Figure 9. Caspase 3 and Fas can act independently to induce cell death, and this may explain why infected WT animals have a modest increase of apoptotic cells in spite of a decrease in caspase 3. It also may suggest that the increased apoptosis in OPN KO is a result of both caspase 3 and Fas combined actions. We are proposing that OPN is a key regulator of a caspase 1-mediated control, upstream this process, orchestrating the balance between caspases 1 and 8. However, alternative mechanisms of an OPN-mediated control of cell death have been proposed. For instance, Suzuki and collaborators have shown that an X-linked apoptosis inhibitor has an ubiquitin-protein ligase activity degrades caspase 3, conferring an antiapoptotic effect in Fas-mediated cell death [30]. The role of OPN in ubiquitin-mediated degradation of protein targets has been suggested in other models [31–33]. Whether ubiquitination is a mechanism by which OPN affects caspase 3 levels and activity remains to be examined. Alternatively, Graessman and colleagues have proposed that OPN has antiapoptotic effects by affecting a target downstream caspase 3 [34], adding another potential layer of OPN-mediated control.

In many models, apoptosis is a defense mechanism against viral infection. On the other hand, some viruses may take advantage of apoptosis for spreading, especially in neurons. This may be the case for the WNV infection of the CNS [35]. Apoptotic pathways are mainly mediated by sequentially activated intracellular cysteine proteases, primarily the executioner caspase 3 [36], which cleaves several substrates and causes DNA breakdown and cell death [37]. Caspase 3 is activated by caspases 8 and 9 [38, 39]. In WNV-infected WT animals, the source of caspase 8 may be the CD95/FADD apoptotic signaling pathway, TNFa signaling, or TLR3 stimulation. However, changes in FADD and CD95 in the infected brains were not affected by OPN. Thus, further studies are necessary to determine the specific mechanism by which OPN upregulates caspase 8 in the WNV-infected brain. As caspase 8 was not increased in the absence of OPN, our data supports that OPN is a negative regulator of a caspase 1-mediated, caspase 8-independent pathway that increases caspase 3 [40] and inflammasome components. The rapid induction of acute innate immune cytokines in OPN KO mice may explain the switch from caspase 8 to caspase 1/3 and the subsequent inflammatory and apoptotic responses (Figure 9).

It is known that caspase 1 has an additional role to cleave and activate IL1b and IL18 at the functional level [41–43]. Recently, it has been shown that caspase 8 is also able to produce IL1b and IL18 independently of caspase 1 [44]. Even though transcriptional increase of IL1b and IL18 levels was similar between the two groups of animals infected with WNV, the cleaved and active form of IL1b was more abundant in OPN KO mice in which caspase 1 levels were higher. This result confirms a role for OPN as a negative regulator of caspase 1-mediated inflammasome.

The enhanced inflammatory response in the brain of infected OPN KO mice is detectable by the increased expression of Iba-1, a microglial marker, suggesting a role for these cells as responders but also as potential targets of OPN regulation. The fast increase in microglial inflammasome has been previously correlated with CNS pathology and dysfunction in other viruses that enter the brain, such as in HIV [45, 46].

Here, we show that, in the absence of OPN, CNS viral infections lead to an acute cytokine upregulation that promotes caspases 1 and 3 and modifies the microenvironment to cause apoptosis and exacerbate inflammation. Whether caspase activation is directly involved in WNV mortality is not known. Our results suggest that OPN plays an important role in the CNS early after viral infection. OPN may prevent a rapid rise of inflammatory factors. This could be missed in studies performed at later time points. Together, these mechanisms may participate in protection by slowing viral spread by preventing apoptosis in the CNS and critically controlling inflammation.
